# Uncovering Adiponectin Replenishing Property of Sujiaonori Algal Biomaterial in Humans

**DOI:** 10.3390/md15020032

**Published:** 2017-02-08

**Authors:** Nlandu Roger Ngatu, Mitsunori Ikeda, Hiroyuki Watanabe, Mamoru Tanaka, Masataka Inoue, Sakiko Kanbara, Sayumi Nojima

**Affiliations:** 1 Graduate School of Health & Nursing Sciences, University of Kochi, Kochi 781-8515, Japan; mikeda@cc.u-kochi.ac.jp (M.I.); inoue@cc.u-kochi.ac.jp (M.I.); kanbara@cc.u-kochi.ac.jp (S.K.); nojimas@cc.u-kochi.ac.jp (S.N.); 2 Faculty of Nutrition, University of Kochi, Kochi 781-8515, Japan; watana@cc.u-kochi.ac.jp (H.W.); m-tanaka@cc.u-kochi.ac.jp (M.T.)

**Keywords:** adiponectin, BMI, blood pressure, dietary supplementation, Sujiaonori

## Abstract

The replenishment of adiponectin—an adipocyte-derived hormone with salutary health effects—has recently been proposed as a new approach to treat hypertension, also ameliorate cardiovascular and metabolic risks. We conducted a prospective placebo-controlled, non-randomized and investigator-blinded dietary intervention study to evaluate the health effects of dietary intake of Sujiaonori (*Ulva/Enteromorpha prolifera Müller*) algal biomaterial (SBM), especially on adiponectin production, blood pressure (BP), and body mass index (BMI) in human subjects. Participants (*N* = 32) were divided into two equally sized groups (*n* = 16 for each group): SBM group (subjects supplemented with 3 g SBM powder twice a day during meal) and the control group (subjects who took 3 g of a supplement made of 70% corn starch powder and 30% spinach twice a day) for four weeks. Two health survey questionnaires (dietary and current health questionnaires) were completed anonymously, saliva sampling was done for adiponectin measurement by ELISA, and blood pressure (BP) and anthropometric parameters were measured at baseline and four weeks later. Student paired *t*-test was performed to compare baseline and post-intervention data on outcome variables between the two study groups. Results showed a 2.24-fold increase in adiponectin level in SBM group (2.81 and 6.26 ng/mL at baseline and at the end of study, respectively) (*p* < 0.01); whereas no significant change was observed in controls (3.58 and 3.51 ng/mL, respectively) (*p* > 0.05). In SBM subjects, an improvement of BP profile was noted with a significant decrease in systolic BP (*p* < 0.01). A positive correlation was found between SBM supplementation and adiponectin level, whereas an inverse correlation was noted between SBM supplementation and blood pressure, and also BMI. These findings suggest that SBM-increased adiponectin level and improved BP in a sample of Japanese young adults, and has the potential to improve blood pressure in humans.

## 1. Introduction

Adiponectin—an adipocyte-derived secretory factor that promotes insulin sensitivity and cell survival—is reported to decrease inflammation, regulate energy homeostasis, and exert salutary activities on cardiovascular health [1,2]. A previous study by Okamoto and colleagues found that adiponectin has an antiatherogenic effect [3], and other studies have associated adiponectin deficiency with diabetes, hypertension, atherosclerosis, coronary heart disease (CHD), and cerebral vascular diseases [4,5,6]. In addition, a number of clinical studies have suggested that adiponectin has a protective effect against cancer [7,8]. This cluster of diseases could be alleviated through pharmacological interventions that restore the decreased adiponectin levels. Thus, researchers have been attempting to find compounds that would produce similar biological effects as adiponectin or increase the endogenous adiponectin [9,10,11,12]. Recent animal experiments on AdipoRon—a newly discovered adiponectin receptor (AdipoR) agonist with similar effects as adiponectin—have suggested that it could be a promising therapeutic agent for the treatment of obesity-related diseases such as type-2 diabetes and cardiovascular disorders [13,14].

Sujiaonori—the Japanese name for *Enteromorpha prolifera Müller* [15,16], which belongs to the family of *Ulvacea* in the order of *Ulvales*—is an edible green river alga that grows in Shimanto River, in Kochi, Japan. This algal species is rich in fiber (60%–65% dry weight), contains bioactive compounds (polyphenols, polyunsaturated fatty acids, vitamins), and is reported to have antioxidant, anti-inflammatory, immunostimulatory, and hypolipidemic properties [17,18,19,20]. We conducted a dietary intervention study and an experimental study on the effects of Sujiaonori-based supplement on adiponectin level, cardiovascular health, the gastrointestinal system, and a number of chronic inflammatory disorders. In this study, saliva—which has recently been used as an alternative non-invasive bio-specimen in clinical studies related to cardiovascular and metabolic disorders [21]—was used for adiponectin measurement. The present report highlights findings related to the effects of dietary intake of Sujiaonori algal biomaterial (SBM) on adiponectin, blood pressure (BP), and body mass index (BMI) in apparently healthy human subjects.

## 2. Results and Discussion

### 2.1. Characteristics of Participants

Of the 38 subjects enrolled, 32 completed the study, including 27 (84.4%) nursing students (age range: 20–29 years) and five (15.6%) teaching staff (age range: 31–54 years). The brief self-administered diet history questionnaire (BDHQ) survey questionnaire used in our study revealed the daily intake of different nutrients by the study participants. The estimated daily calorie intake for the SBM group was 1303.9 ± 478.9 kcal and 1480.8 ± 630.1 kcal before and after dietary intervention, respectively, and it was 1284.1 ± 461.5 kcal and 1315.6 ± 391.8 kcal in controls (*p* > 0.05). Except for alcohol intake (which was reduced in each group during the study period), no significant difference was observed with regard to the daily intake of nutrients within each group, and also when comparing SBM subjects to controls (*p* > 0.05). The analysis of baseline blood pressure (BP) indicated that six subjects (three SBM, three controls subjects; 18.7%) had a personal history of hypertension, 10 (six SBM subjects, four controls; 32%) had either prehypertension (systolic BP: 120–139 mmHg) or high blood pressure (systolic BP: 140 mmHg or higher). On the other hand, nine subjects (28%) had a baseline diastolic BP between 80 and 89 mmHg or higher, including four SBM subjects and five controls. Assessment of the BMI profile of participants revealed that two (6.2%) SBM subjects were overweight (BMI ≥ 25 kg/m^2^); all other subjects (93.8%) had a BMI < 25 kg/m^2^.

### 2.2. SBM Supplementation Induces Adiponectin Replenishment

**Figure 1:**
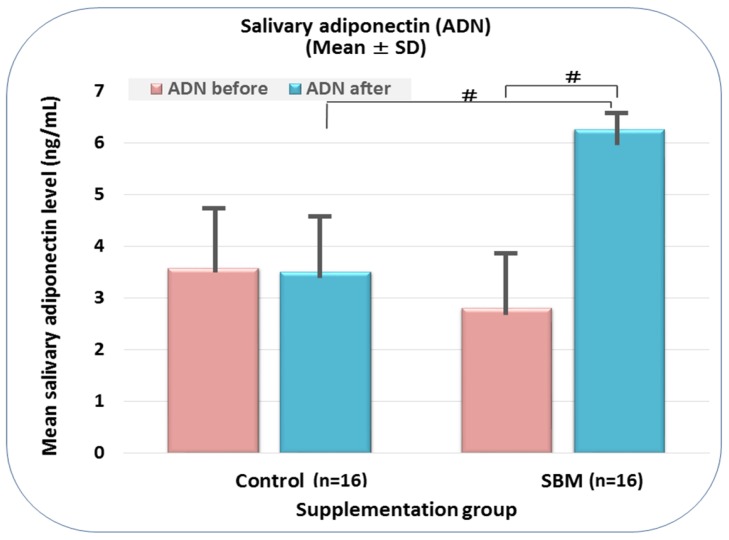
Distribution of salivary adiponectin according to study group. Legend: #, *p*-value less than 0.01 versus baseline value as well as controls; ADN, adiponectin; SD, standard deviation. Figure 1 shows an increase of adiponectin level within Sujiaonori algal biomaterial (SBM) group (vs. baseline values) and also when compared to the Control group (*p* < 0.01).

As shown in Figure 1, a 2.24-fold increase of salivary adiponectin level was observed in the SBM group, from 2.81 at baseline to 6.26 ng/mL and four weeks later (*p* < 0.01), whereas no significant difference was noted in the control group (3.58 ng/mL and 3.51 ng/mL, respectively) (*p* > 0.05). In addition, when both study groups were compared, a markedly higher adiponectin level was observed in the SBM group (*p* < 0.01; vs. control).

The effects of Sujiaonori on human health have been rarely studied. Animal experiments on sulfated polysaccharides (ulvans) or ulvan fractions from several *Ulva* species have indicated promising health benefits [22,23]. However, the effects of Sujiaonori on cardiovascular health have not been investigated. Our preliminary study showed that SBM supplementation increased adiponectin level close to the highest end of normal value in humans (6.42 ng/mL) [24]. The administration of exogenous adiponectin as a drug is proposed as an alternative therapeutic approach in the management of cardiovascular and metabolic disorders associated with adiponectin deficiency. Furthermore, other studies have suggested that the discovery of novel pharmacological agents that increase adiponectin level or the development of adiponectin agonists could be an important step in the treatment of these conditions [25,26].

Obviously, natural compounds able to increase the endogenous adiponectin may assist in the prevention of disease initiation in at-risk subjects or may alleviate cardiometabolic disorders [27]. For example, previous studies have shown that daily intake of *eicosapentaenoic acid* (EPA)—one of the omega-3 polyunsaturated fatty acids (PUFA) that are abundant in marine fish meat and oil—induced an increase in adiponectin level in mice [28]. In addition, there are a few natural compounds and plant materials that have been reported to influence adiponectin level. For example, a combination of *docosahexaenoic acid* (DHA, another PUFA) and canola oil [10], as well as compounds from the medicinal herb *Radix astragali* (such as *astragaloside* II and *isoastragaloside* I [29]) have increased adiponectin level in animal experiments. Another study by Williams et al. [30] showed that coffee consumption upregulated adiponectin production; however, this finding was controversial, as other researchers have found that daily intake of coffee did not influence adiponectin level in healthy or sick Japanese men and women [31]. In our study, a 2.24–2.96-fold increase of adiponectin level was observed, and no adverse effects were reported by the SBM subjects. Future comparative clinical investigations could possibly provide knowledge on the optimum doses of Sujiaonori-based supplement that could be beneficial in humans. Furthermore, current smoking is reported to decrease adiponectin, and this reduction can be reversed by quitting smoking [32,33]. In our study, no participant smoked, and the intake of fish products and dietary fiber did not vary within the study period. Overall, no significant differences were noted in terms of nutrients intake between the study groups, which suggested that the observed changes in adiponectin level and blood pressure profile were attributable to the supplements used in this preliminary study.

### 2.3. Effects of SBM Supplementation on BMI, Blood Pressure, and Correlation between Salivary Adiponectin and Outcome Parameters

**Figure 2:**
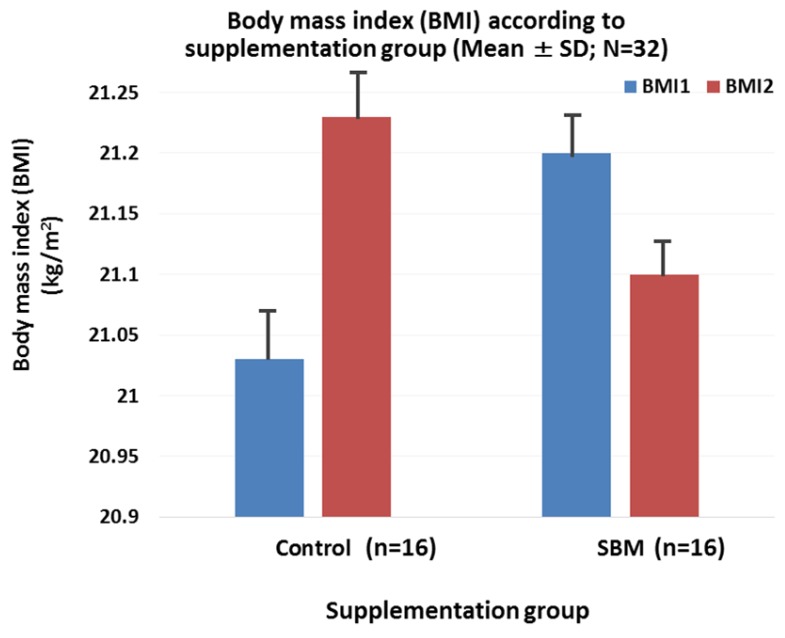
Trend of body mass index (BMI) according to study group (*N* = 32). Legend: BMI, body mass index; BMI1, value of body mass index before intervention; BMI2, value of body mass index after intervention; *N*, sample size; SD, standard deviation; SBM, Sujiaonori algal biomaterial. The figure shows a slight reduction of BMI in the SBM group, but the difference was not significant when comparing BMI values before and after SBM supplementation, or control and SBM groups (*p* > 0.05).

Figure 2 shows the trend in BMI in each of the study groups before (baseline) and after a 4-week intervention. Though a relatively lower BMI observed in the SBM group and higher BMI in controls at the end-of-study evaluation, no statistically significant change was observed within each group and between the study groups compared to baseline BMI values (*p* > 0.05).

**Figure 3:**
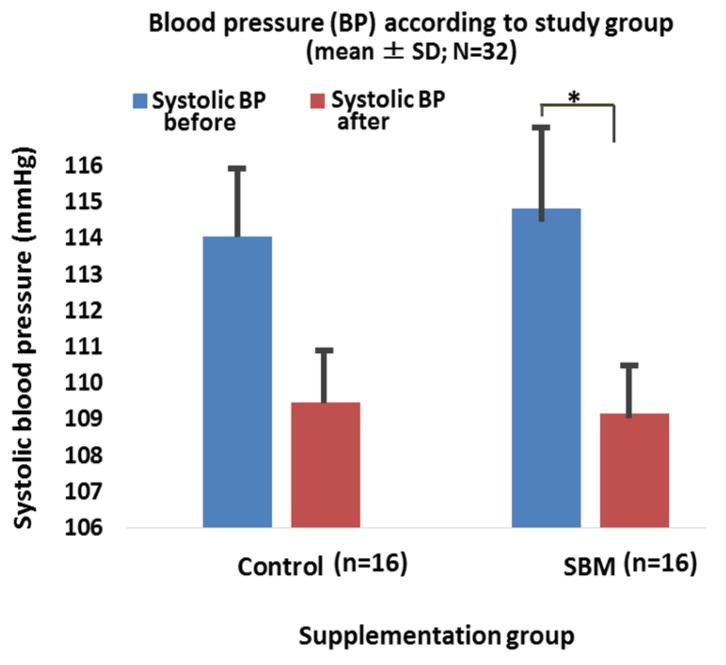
Distribution of blood pressure according to supplementation group. Legend: *, *p*-value less than 0.05 versus baseline value in SBM group; SD, standard deviation; SBM, Sujiaonori algal biomaterial; BP, blood pressure. The figure shows the reduction of systolic BP in both study groups, with a significant difference in the SBM group (*p* < 0.05), whereas a marginally significant difference was noted in controls (*p* = 0.0611). In controls, a reduction of systolic BP was observed, but not significantly (*p* > 0.05). No difference was observed when comparing SBM and control groups (*p* > 0.05).

Regarding blood pressure, after a 4-week supplementation period, a reduction in systolic BP was observed within each of the study groups. Regarding the systolic BP, a statistically significant decrease was observed in the SBM group (*p* < 0.05), whereas a marginally significant reduction was noted in the control group (*p* = 0.0611). When comparing both study groups, no significant change was observed (*p* > 0.05) (Figure 3). In addition, results from this preliminary study also showed a reduction of systolic BP in SBM subjects with a baseline systolic BP of 120 mmHg or higher, including subjects with prehypertension (*p* < 0.01, not shown). Regarding diastolic BP, a decrease was observed in each of the study groups, but not significantly (*p* > 0.05, not shown).

Hypertension is considered to be the greatest contributor to carotid arteriosclerosis in the Japanese population [34]. Numerous meta-analyses of large-scale clinical trials have shown that prehypertension increased cardiovascular risk [35]. Our study showed a reduction of systolic BP in the SBM group, and a positive correlation between SBM supplementation and adiponectin level. In the SBM-supplemented group, adiponectin level was inversely correlated with BMI, and positively with age. No association was found between adiponectin level and other clinical parameters such as diastolic BP and physical activity (not shown).

To summarize, our study showed that the intake of SBM supplement increased adiponectin level and improved BP in subjects with either prehypertension or high BP at baseline. The latter effect might be attributable mainly to the increased adiponectin level. Considering the beneficial health effects of adiponectin in humans [1,5], and given that the upregulation of adiponectin production is reported to ameliorate cardiovascular risk such as prehypertension and hypertension [36], a food product or supplement that can replenish adiponectin might be beneficial in reversing the disease course of patients with those conditions or eliminating the disease risk in individuals at risk. Thus, SBM has the potential to ameliorate blood pressure and related cardiovascular risks. However, despite the health effects observed, our study has some limitations; it was conducted in young subjects who were apparently healthy, and findings might differ in older subjects. Investigating the effects of SBM supplementation in subjects from different age-groups as well as in individuals with documented cardiovascular and/or metabolic disorders may provide more information on the magnitude of the health benefits of Sujiaonori.

## 3. Materials and Methods

### 3.1. Study Design, Participants, and Survey Questionnaires

**Figure 4:**
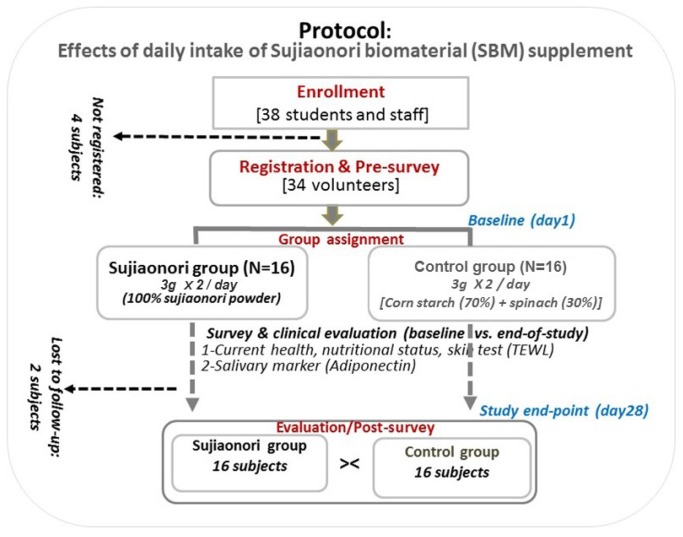
Clinical trial protocol on the effects of daily dietary intake of Sujiaonori supplement adiponectin secretion and cardiovascular health. Legend: TEWL, transepidermal water loss.

We conducted a non-randomized, placebo-controlled, investigator-blinded dietary intervention on the health effects of SBM dietary intake from January through August 2016 in Kochi prefecture, Japan. The participants were adult nursing students and teaching staff from the University of Kochi who satisfied the following inclusion criteria: 20 years of age or older, absence of a major health condition, taking no medication, voluntary enrollment, and provision of a signed informed consent form. Individuals with a personal and/or family history of allergy to algal product, ongoing medication, having heart, liver, major brain or kidney disease were not eligible. Of the 38 volunteers who were enrolled; 34 got registered to confirm their participation and received survey sheets, of whom 32 completed the study, including 31 women and 1 man. Two participants who did not undergo the second clinical examination and saliva sampling were excluded for the analysis related to this report (Figure 4).

Participants were registered and assigned to study groups using their identification numbers; the SBM group comprised odd-numbered subjects who had to take 3 g SBM powder twice daily during mealtimes, control group was even numbered-subjects who received a 3 g mixture of 70% corn starch and 30% Japanese spinach powder twice daily. Dried Sujiaonori and corn starch samples were provided by a local food processing factory. The experimental period was four weeks. SBM powder and control product were prepared at our food science laboratory in the University of Kochi.

At baseline and four weeks later, the “brief self-administered diet history questionnaire” (BDHQ) and the Current Health Status Questionnaire (CHSQ) were anonymously completed by participants, and anthropometric parameters and blood pressure were measured. In addition, saliva samples were collected at baseline and on day 28 of the study for the measurement of salivary adiponectin. BDHQ—which was used to assess the dietary history of participants—is a four-page fixed-portion questionnaire that asks about the intake of 58 selected food and beverage items in the previous month (BDHQ questionnaire, University of Tokyo) [37]. CHSQ was prepared by our research team, and comprised questions related to sociodemographics (gender, age, and occupation), lifestyle-related information (smoking habit, alcohol consumption, and physical exercise), intake of Sujiaonori or other algal food product, history of hypertension or hypotension, and other health conditions.

### 3.2. ELISA Assay and Outcome Variables

For saliva sampling, whole saliva (approximately 5 mL) was collected in tube with a funnel at our laboratory between 8:00 and 9:00 am, at least 60 min prior to brushing teeth, eating, or drinking. Test tubes were placed on ice just after saliva collection and stored at −20 °C. Salivary adiponectin was assayed by Enzyme-linked Immunosorbent Assay (ELISA) using a Human Adiponectin ELISA Kit (AssayPro, St Charles, MO, USA) in accordance with the manufacturer’s instructions. Briefly, collected samples were centrifuged at 800× *g* for 10 min. Reagents, standards, and saliva samples were prepared at room temperature. First, the excess microplate strips were removed from the plate, and 50 μL of adiponectin standard or saliva sample was added to each of the wells. Then, the wells were covered with a sealing tape and the plate was incubated for 1 h.

After each well was washed with washing buffer (200 μL), 50 μL of conjugate was added, and the plate was incubated for 30 min. After incubation, the microplate was washed as previously described. Fifty microliters of chromogen substrate were added to each well, and a 10 min incubation was performed. Next, 50 μL stop solution was added to each well, and the color changed from blue to yellow. The absorbance of the microplate was measured at a wavelength of 450 nm. The mean values of the triplicate readings for each standard and sample were calculated. A standard curve was generated, and the graph was plotted with the standard concentrations on the *x*-axis and the corresponding mean 450 nm absorbance on the *y*-axis. The essay was performed in duplicate. The primary outcome variable was the salivary adiponectin level, and the secondary outcome variables were blood pressure (BP), BMI, and health outcomes retrieved from the answers to survey questionnaires. In this paper, only data on salivary biomarker and a selected number of cardiovascular risk parameters (blood pressure, history of hypertension, hypotension, and BMI) are reported. Prehypertension (systolic BP: 120–139 mmHg; diastolic BP: 80–89 mmHg) and hypertension (systolic BP >139 mmHg; diastolic BP >89 mmHg) were defined according to the American Heart Association [38].

### 3.3. Ethics Approval and Consent to Participate

Ethical approval of the study protocol was obtained from the ethics committee of the faculty of Nutrition, University of Kochi (Approval reference: No15-10, November 2015). The study protocol was registered in an international trial registry (ISRCTN35616776). Additionally, each participant provided a signed informed consent. This research was conducted in accordance with the rules of the Declaration of Helsinki of 1975, as revised in 2008.

### 3.4. Data Analysis

Stata software version 13 (Stata Corp, College Station, TX, USA) was used for statistical analyses. Continuous outcome variables are described as means or percentages, and comparison between study groups was performed with the use of analysis of variance (one-way ANOVA). For binary variables, data are described as proportions. A Spearman correlation test was performed to assess the association between salivary adiponectin, age, occupation, blood pressure, and BMI; on the other hand, multivariate logistic regression test was used to assess the association between salivary adiponectin and categorical variables (physical exercise, history of intake of algal food product, history of hypertension or hypotension). Statistical significance for *p*-values was set at 0.05.

## 4. Conclusions

Findings from this study suggested that the daily dietary intake of SBM supplement replenished adiponectin and ameliorated BP in subjects with prehypertension. Thus, Sujiaonori represents a source of promising bioactive materials that have a potential to improve blood pressure.

## Abbreviations

The following abbreviations are used in this manuscript:

BDHQ	brief self-administered diet history questionnaire
BMI	body mass index
BP	blood pressure
CHD	coronary heart disease
DBP	diastolic blood pressure
ELISA	enzyme-linked immunosorbent assay
SBM	Sujiaonori biomaterial
SBP	systolic blood pressure
